# The Roles of Trust in Government and Sense of Community in the COVID-19 Contact Tracing Privacy Calculus: Mixed Method Study Using a 2-Wave Survey and In-Depth Interviews

**DOI:** 10.2196/48986

**Published:** 2024-03-07

**Authors:** Hyunjin Kang, Jeong Kyu Lee, Edmund WJ Lee, Cindy Toh

**Affiliations:** 1 Wee Kim Wee School of Communication and Information Nanyang Technological University Singapore Singapore; 2 Department of Health and Exercise Science University of Oklahoma Norman, OK United States; 3 Department of Anthropology Columbia University New York, NY United States

**Keywords:** COVID-19, contact tracing technology, privacy calculus, trust in government, sense of community, mixed method, mobile phone

## Abstract

**Background:**

Contact tracing technology has been adopted in many countries to aid in identifying, evaluating, and handling individuals who have had contact with those infected with COVID-19. Singapore was among the countries that actively implemented the government-led contact tracing program known as TraceTogether. Despite the benefits the contact tracing program could provide to individuals and the community, privacy issues were a significant barrier to individuals’ acceptance of the program.

**Objective:**

Building on the privacy calculus model, this study investigates how the perceptions of the 2 key groups (ie, government and community members) involved in the digital contact tracing factor into individuals’ privacy calculus of digital contact tracing.

**Methods:**

Using a mixed method approach, we conducted (1) a 2-wave survey (n=674) and (2) in-depth interviews (n=12) with TraceTogether users in Singapore. Using structural equation modeling, this study investigated how trust in the government and the sense of community exhibited by individuals during the early stage of implementation (time 1) predicted privacy concerns, perceived benefits, and future use intentions, measured after the program was fully implemented (time 2). Expanding on the survey results, this study conducted one-on-one interviews to gain in-depth insights into the privacy considerations involved in digital contact tracing.

**Results:**

The results from the survey showed that trust in the government increased perceived benefits while decreasing privacy concerns regarding the use of TraceTogether. Furthermore, individuals who felt a connection to community members by participating in the program (ie, the sense of community) were more inclined to believe in its benefits. The sense of community also played a moderating role in the influence of government trust on perceived benefits. Follow-up in-depth interviews highlighted that having a sense of control over information and transparency in the government’s data management were crucial factors in privacy considerations. The interviews also highlighted surveillance as the most prevalent aspect of privacy concerns regarding TraceTogether use. In addition, our findings revealed that trust in the government, particularly the perceived transparency of government actions, was most strongly associated with concerns regarding the secondary use of data.

**Conclusions:**

Using a mixed method approach involving a 2-wave survey and in-depth interview data, we expanded our understanding of privacy decisions and the privacy calculus in the context of digital contact tracing. The opposite influences of privacy concerns and perceived benefit on use intention suggest that the privacy calculus in TraceTogether might be viewed as a rational process of weighing between privacy risks and use benefits to make an uptake decision. However, our study demonstrated that existing perceptions toward the provider and the government in the contact tracing context, as well as the perception of the community triggered by TraceTogether use, may bias user appraisals of privacy risks and the benefits of contact tracing.

## Introduction

### Background

Contact tracing technology has been implemented in many countries to help identify, assess, and manage individuals exposed to persons infected with COVID-19 [1]. The main aims of a digital contact tracing system are not only to notify those who may have contracted the virus unknowingly but also to provide corresponding information and instructions from public health officials. Hence, some of contact tracing applications are designed to track the geolocations and mobility patterns of individuals using GPS and location-based solutions [2,3]. Digital contact tracing identifies individuals’ close contacts with people confirmed or suspected of having COVID-19 through Bluetooth technology [4] or even allows authorities to make decisions as to whether certain individuals are certified as healthy enough to enter public spaces [5]. The use and implementation of contact tracing apps during COVID-19 brings concerns of privacy intrusion and surveillance to the forefront, as the attempt to digitally map and trace the spread of the virus is conducted through apps that collect some of the most personal and sensitive data. Thus, privacy concerns were identified as a significant barrier to adopting the contact tracing system, significantly influencing the intention to participate in the program [6,7].

Applying the privacy calculus model, that is, the extent to which individuals are willing to disclose personal information after weighing the risks and benefits, this study examines how individuals’ perceptions of two important key entities—government and community members—involved in digital contact tracing play roles in their privacy calculus for adopting contact tracing. First, most contact tracing systems, including those in Singapore, Ireland, and France, were introduced and managed by the national government [6]. Derived from the social exchange concept, prior research on privacy suggests that trust in a service provider is a significant determinant of privacy calculus on the web [8,9]. Considering the managerial roles played by the government for contact tracing measures and the extensive personal information collected and analyzed for this effort, we postulated that individuals’ trust in the government would be the key influencer of their privacy calculus in digital contact tracing.

Community members are another key entity involved in the privacy calculus in digital contact tracing. The active participation of community members in the contact tracing program is crucial for the success of preventive efforts. However, digital contact tracing implies that the participants are connected through a web-based system for tracking, which may trigger privacy risks among users. Therefore, the sense of community triggered by the contact tracing measure may influence the relationship between privacy concerns and benefits, shaping one’s intention to participate in contact tracing efforts.

This study focuses on understanding privacy calculus in the context of using TraceTogether, a nationwide contact tracing program implemented in Singapore during the COVID-19 pandemic. The Government Technology Agency of Singapore, also known as GovTech, launched TraceTogether on March 20, 2020, followed by the launch of a token on June 28 to increase accessibility for those without a smartphone. In late December 2020, it became mandatory to check using TraceTogether before entering various places, including restaurants, workplaces, schools, places of worship, and shopping malls. As COVID-19 prevention measures eased, TraceTogether check-ins were no longer required in most settings from April 26, 2022.

### Objective

This study investigates how trust in government and the sense of community during the early stage of contact tracing influence privacy calculus and intention to use the technology during the later stage when contact tracing becomes compulsory. To this end, a 2-wave survey and a follow-up in-depth interview with those who participated in the contact tracing program in Singapore were conducted. Building on the survey results, in-depth interviews were conducted to gain insights into privacy calculus in digital contact tracings, as well as the ways and reasons why trust in government and sense of community impact privacy calculus in contact tracing for COVID-19 prevention.

### Literature Review

#### Theoretical Framework: Privacy Calculus Theory

##### Overview

Privacy concerns about contact tracing programs during the COVID-19 pandemic have been raised because of the potential risks of extensive data collection, surveillance, and the possibility of unauthorized access [6,7], necessitating a careful balance between public health benefits and safeguarding individuals’ private information. Thus, privacy calculus theory has been used as one of the most robust theories within the field of communication [10] to unpack the underlying theoretical mechanisms in explaining the use of contact tracing apps through the lens of privacy. Having its theoretical roots and origins in economics and social exchange theory, individuals are assumed to be rational in making decisions and weighing trade-offs, and these decisions can be mathematically modeled as a function of the interaction between the benefits and risks of adopting a behavior [11]. For these reasons, privacy calculus theory offers a useful framework for investigating the influence of competing positive and negative beliefs on individuals’ willingness to engage with technologies by capturing the extent of trade-offs individuals are willing to make in relation to privacy [12]. For instance, despite knowing that companies such as Google or Waze could identify individuals’ precise geocoordinates, individuals who commute by driving may be willing to disclose this otherwise sensitive information (where they are and their mobility patterns) in exchange for the benefit of getting to their desired locations.

##### Privacy Concerns About Digital Contact Tracing

At the heart of privacy calculus theory is the act of self-disclosure of personal data, which is a product of two opposing forces: privacy concerns and the perceived benefits of information disclosure. *Privacy concerns* can be defined as individuals’ fears and apprehensions over the possible loss of privacy, as well as how their personal data could be used or abused [13,14]. Privacy concerns are multidimensional by nature, for example, [15,16]. Privacy concerns encompass 3 dimensions: *perceived surveillance*, *perceived intrusion*, and *secondary use of information* [16]. At a fundamental level, privacy concerns are the worries that people are being watched. *Perceived surveillance* encapsulates this notion and refers to individuals’ perceptions of the act of data collection pertaining to their lives—whether legitimate or illegal [17]. In the digital age, this could take the form of devices and technological platforms that capture individuals’ personal activities by recording their behaviors [18]. For instance, people living in Florida expressed concerns over the Florida Department of Health’s Healthy Together contact tracing app in collecting data such as contact lists, phone numbers, and medical conditions [19]. Relatedly, *perceived intrusion* is another dimension of privacy concerns and refers to the extent to which others are able to make independent decisions about possessing or soliciting information. For instance, in China, the government implemented a QR code system for contact tracing and assigned colors, such as green, yellow, or red, depending on the health status of individuals and the risk of exposure by triangulating data from public transport and health care systems [20], which determined whether they had access to public places [21]. The third dimension of privacy concerns is the secondary use of information, defined as the use of personal data collected from individuals for purposes other than what it was intended for [22]. As a large volume of data were collected by contact tracing apps, there were fears over how the government would use (or abuse) potentially sensitive data [20,21].

In the context of contact tracing app use, a recent study showed that when people are concerned about the extent of surveillance and intrusion of the implementation of contact tracing apps, this may negatively influence their intention to use, as fears of public health surveillance could be transformed into routine monitoring of populations extending beyond the purpose of infectious disease management [23]. This is consistent with prior evidence of privacy concerns and different facets of technology use or behaviors to engage in privacy protection behaviors [13,24]. In Singapore, after the government announced that police would have access to TraceTogether data for criminal investigations and after correcting perceptions that the data were only collected for contact tracing and no other purpose, some members of the public expressed concerns and disappointment and indicated their intention to use it less [25].

##### Perceived Benefits

In this study, perceived benefits were conceptualized as the health benefits that individuals would receive by using contact tracing apps, such as protecting them from COVID-19, making them more informed, or improving the overall public health capacity in managing and keeping infections low [12]. Hence, the use of contact tracing during the COVID-19 pandemic addresses a common problem by providing a crucial tool for communal health management, where the benefits extend beyond individual interests.

Although the loss of personal privacy is a major concern for different populations during the use of COVID-19 contact tracing apps, some have recognized the potential benefits of digital surveillance. First, advocates of “big data systems” would argue that tracking and mapping the spread of infectious diseases through digital means (eg, electronic health records) is more efficient than the traditional means of calling and asking people to remember their close contacts during the day and would reduce the burden on health care workers [26]. The data collected could enable public health agencies to quickly map potential hotspots in real time [27] and take decisive actions to curb the spread of COVID-19. Second, the use of contact tracing technology may help users feel empowered. Research has shown that individuals may gain psychological relief and peace of mind by using contact tracing apps as they provide a sense of empowerment and certainty—having data and knowledge about whether they were in close proximity with the infected, as they coped with the ambiguity of the pandemic by monitoring and interacting with their own health data [19]. A study conducted in Sweden [7] found that the benefits of contact tracing apps were positively associated with willingness to use them. They found that the perceived prosocial usefulness of contact tracing apps was positively associated with willingness to use contact tracing apps. Research has also underscored the prevalence of the privacy paradox that, despite concerns over privacy loss, people are willing to accept and use apps as long as they are perceived to be beneficial in lowering infection rates [7].

#### Trust in Government and Sense of Community as Antecedents of Privacy Calculus in TraceTogether

##### Overview

Communication privacy management theory, built upon the privacy calculus model, uses the metaphor of “boundaries” to explain the motivation to reveal or withhold information [28]. According to this theory, boundary openness or closure for information flow is largely dependent on how individuals perceive institutional privacy assurances. In other words, the cognitive processes of risk control assessment and privacy concerns are context specific. Thus, privacy-relevant beliefs are “better related to individuals’ own information experiences and social contexts rather than regarded as a global consequence of technology use per se” [16]. This approach aligns with the notion that privacy is a dynamic process [29,30]. This study also investigated the antecedents that shape privacy calculus in the context of digital contact tracing.

Specifically, we posit that the service provider (ie, government) of the tracing system and other community members who use the device to collectively fight against COVID-19 would be the key entities that would influence individual users’ intentions to use a contact tracing device. This study examines how (1) trust in government and (2) the sense of community triggered by the use of contact tracing technology are associated with one’s privacy calculus regarding digital contact tracing, thereby shaping one’s intention to adopt digital tracing technology.

##### Trust in Government

Social exchange theory suggests that users disclose personal information to gain intangible benefits from a relationship, given that these perceived benefits outweigh the perceived risks [31]. The benefits of a social exchange might differ from an economic exchange in that the former may rely on existing social ties and involve intangible values, including emotions and social power [31]. Similar to other contact tracing programs, TraceTogether is government-initiated. Therefore, user acceptance and adoption are deeply linked to Singaporeans’ trust and confidence in their government [26].

Trust is established when individuals or groups harboring positive perceptions of one another enable the relationship to achieve anticipated results [32]. When an individual places trust in another person, group, or organization, they find themselves liberated from the burden of anxiety and obligation to constantly monitor the actions of the other party [33]. Thus, it can be expected that people who use the contact tracing system will perceive social exchange as more reliable if they trust the government. Supporting this notion, prior studies on web-based privacy have demonstrated that trust in web-based service providers or vendors significantly influences privacy management decisions by decreasing privacy concerns [8,9]. A study in Singapore [34] found that political trust could mediate Singaporeans’ privacy concerns, and those who exhibited trust in their government tended to have more positive attitudes toward digital contact tracing technology. This finding is echoed in multiple studies on contact tracing apps in countries including France [35], Japan [36], Germany [37], and the United Kingdom [38].

Trust in service providers can also be critical to increasing users’ perceived disclosure benefits [39]. Building on the concept of privacy calculus [40], one study [41] argues that gaining users’ willingness to disclose personal information requires the exchange to be based on a fair social contract. In other words, users find the benefits more attractive when they perceive the provider as trustworthy, leading to a positive bias in their privacy calculus. Taken together, we hypothesized that the effects of trust in the government on privacy calculus in TraceTogether would be as follows:

H1: Trust in government increases the perceived benefit of using contact tracing apps.

H2: Trust in government will decrease privacy concerns when using contact tracing apps.

##### Sense of Community Triggered by Contact Tracing

The effectiveness of the contact tracing program for infectious disease prevention largely depends on the participation rate of individuals in the community. Moreover, recognizing the collective action nature of contact tracing underscores its significance in navigating the social dilemma posed by the virus, emphasizing the shared responsibility in curbing the spread for the greater well-being of the community. To enhance the uptake rate of TraceTogether, one way in which the government has promoted the application to Singaporeans is by framing digital contact tracing as a community effort and responsibility. The TraceTogether interface included various elements designed to encourage feelings of closeness and connectedness with other users. For example, the TraceTogether app indicates the number of activated devices nearby and shows how many Bluetooth signals are exchanged among them. The language used in an official promotional video for TraceTogether also reinforced this, depicting the tool as being for “‘community-driven’ contact tracing suggesting that this is a ‘grassroots’ mechanism of fighting the virus” [42]. Therefore, group membership and collective benefit have been leveraged to encourage user adoption and acceptance of TraceTogether.

However, the contact tracing program connects those in the vicinity via Bluetooth technology to estimate the proximity and duration of encounters, which may also trigger privacy concerns. Communication privacy management theory [28] suggests that privacy management is not just about deciding to disclose or withdraw personal information with others (personal boundaries). When one’s personal information is shared, those who can access it become co-owners of the information, suggesting that privacy management also involves comanaging collective boundaries. Thus, the disruption of the synchronized management of collective privacy boundaries among co-owners is a significant source of privacy risk perception. Although individual participants in the contact tracing program cannot access others’ information directly, contact tracing implies that people encountered may be notified of one’s infection. Moreover, signaling connectivity with others through interface cues may trigger privacy risk heuristics, heightening privacy concerns among users [43].

Collectively, we expect that the perception of “the greater good” for the improved safety of loved ones and the community is a significant factor influencing the privacy calculus of being part of the contact tracing program [44,45]. This study hypothesizes a paradoxical function of the sense of community such that the sense of community triggered by digital contact tracing will enhance perceived benefits while simultaneously presenting privacy concerns at the same time:

H3: The sense of community triggered by contact tracing apps increases the perceived benefits of contact tracing apps.

H4: The sense of community triggered by contact tracing apps will increase the privacy concerns of contact tracing apps.

This study also examined the interplay between one’s perception of the government and the community on privacy calculus in digital contact tracing. Although trust in government has developed over time, a sense of community can be promoted through various design and marketing elements of digital contact tracing. As discussed above, TraceTogether was promoted to Singaporeans by framing contact tracing as a community effort and responsibility [46]. The user interface of the application was also designed to imbue users with a sense of community through various design elements. Thus, the study attempts to test whether one’s trust in government factors in the privacy calculus in contact tracing would vary as a function of the larger community that they perceive through participation in the collective contact tracing effort. However, given the absence of literature documenting the moderating role of sense of community in the relationship between trust in government and privacy calculus, this study investigates the interplay between sense of community and trust in government, proposing the following research question:

RQ1. Will the sense of community generated by contact tracing apps moderate the influence of trust in government on privacy concerns and perceived benefits of using contact tracing apps?

**Figure 1:**
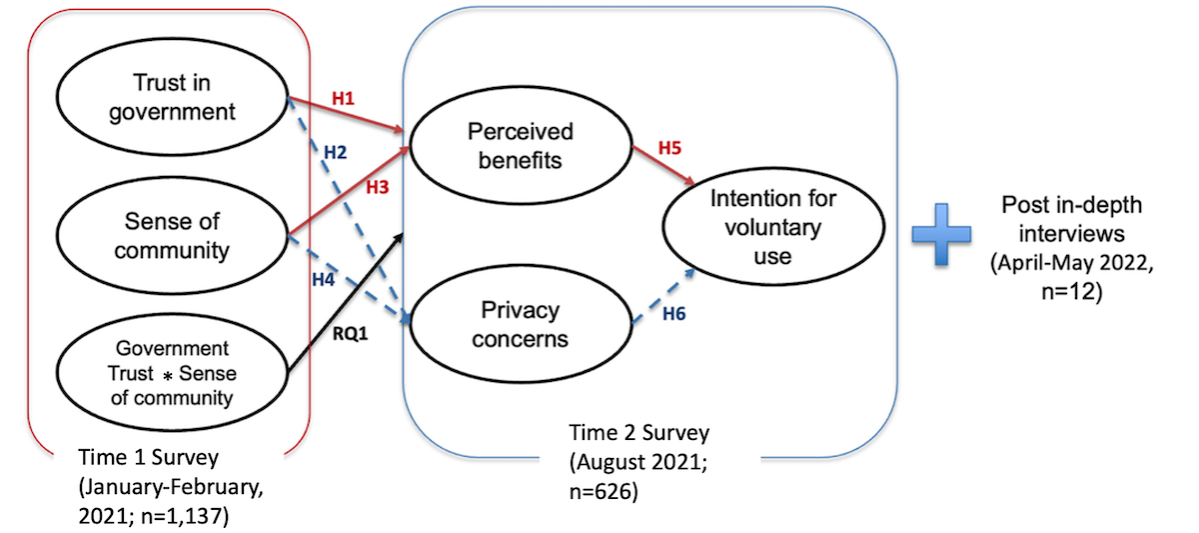
Summary of the study model and methodology. H: hypothesis; RQ: research question.

Finally, based on the basic tenet of privacy calculus theory discussed earlier, we also hypothesize that privacy concerns and perceived benefits will have opposite associations with the intention to use TraceTogether. Given that the use of a contact tracing system itself implies the disclosure of personal information, we tested the intention to use TraceTogether as the outcome of the privacy calculus. Checking through TraceTogether was mandatory to enter most venues, including shopping malls, offices, workplaces, and schools, at the time of the second wave of data collection when use intention had been measured. Therefore, the measurement was designed to assess their intention to use TraceTogether voluntarily until the pandemic ended, rather than merely assessing their intention to use the device for a certain duration. The study model and methodology are illustrated in Figure 1.

H5: Perceived benefits are positively associated with the intention to actively participate in contact tracing programs.

H6: Privacy concerns are negatively associated with the intention to actively participate in the contact tracing programs.

### Study 1: Two–Wave Longitudinal Survey

#### Methods

##### Recruitment

To test our hypotheses and research questions, we used a mixed method approach involving a 2-wave survey and in-depth interviews. The first survey was conducted from January to February 2021 with panel members provided by a local research company. English-speaking Singaporean citizens or permanent residents aged 21 years or above participated in the survey (N=1198). The quota sampling procedure was employed to ensure that the study sample represented the proportion of the ethnic structure of the national population [47]. The first study was conducted in an omnibus survey format to understand the various perceptions and behaviors related to COVID-19 and contact tracing practices. Among all participants who completed the survey, 94.91% (n=1137) responded that they had downloaded the TraceTogether mobile app or collected the token.

A follow-up second-wave survey was carried out in August 2021, approximately 6 months after the first survey. This survey assessed privacy calculus factors: privacy concerns, perceived benefits, and adoption intention. A second-wave survey invitation was sent to those who completed the first survey. In total, 674 participants completed the second-wave survey (response rate: 59.3%). Data from those who either downloaded the TraceTogether mobile app or received the token and who completed both studies at the point of the first wave of data collection were included in the study sample (n=626; see Table 1 for the descriptive statistics of the study sample). Our sample comprised 88.7% (n=555) Chinese, 4.3% (n=27) Malay, and 7% (n=44) Indian individuals. In comparison to the ethnic structure of the national population in Singapore, where Chinese people constitute 74%, Malay people 13%, and Indian people 9% [48], our sample overrepresented the Chinese group and underrepresented the Malay group. Moreover, the sample included only a small portion of participants aged older than 65 years (n=25; 4%), thereby underrepresenting the older population. During the first wave of data collection (January-February 2021), the TraceTogether app or token could be used for checking at selected venues where people were likely to be in close contact for prolonged periods or where high human traffic is expected. These venues included schools, educational institutes, shopping malls, restaurants, and workplaces. People can still choose to scan the QR code displayed at the entrance instead of using TraceTogether. From June 2021, the use of TraceTogether became mandatory when entering these venues.

**Table 1:** Descriptive statistics.

Variables			Values (N=626)
**Gender, n (%)**			
	Male	332 (53)	
	Female	294 (47)	
**Age group (y), n (%)**			
	21-34	130 (20.8)	
	35-49	281 (44.9)	
	50-64	190 (30.3)	
	65 or older	25 (4)	
	Mean age (y), mean (SD)	44.9 (11)	
**Ethnicity, n (%)**			
	Chinese	555 (88.7)	
	Malay	27 (4.3)	
	Indian	44 (7)	
**Education level, n (%)**			
	Secondary and below	13 (2)	
	Preuniversity	270 (43.1)	
	University degree or above	343 (54.8)	
**Yearly household income (Singaporean $^a^), n (%)**			
	<2000	37 (5.9)	
	2000-6000	185 (29.6)	
	6000-10,000	241 (38.5)	
	≥10,000	163 (26)	
**Self-assessed health status, n (%)**			
	Poor	18 (3)	
	Fair	163 (26)	
	Good	293 (46.8)	
	Very good	126 (20.1)	
	Excellent	26 (4)	
**TraceTogether adoption**			
	Have downloaded TraceTogether app (Yes)	Wave 1: 494 (78.9); wave 2: 586 (93.6)	
	Have collected TraceTogether token (Yes)	Wave 1: 452 (72.2); wave 2: 549 (87.7)	

^a^Singaporean $1=US $0.75.

##### Measurement

The two exogenous variables (government trust, sense of community) and control variables (demographic information and TraceTogether use) were measured in the first-wave survey. Privacy calculus factors—privacy concerns and perceived benefits—and TraceTogether use intention were measured in the second-wave survey.

*Trust in government* was measured using 3 items adapted from Wu et al [49]. The *sense of community* was measured using 5 items adapted from 2 studies [50,51]. The sense of community captured the extent to which users felt a sense of belonging and emotional ties with other TraceTogether users. *Privacy concerns* were assessed using 10-item measures which captured 3 aspects of privacy concerns, namely perceived surveillance, perceived intrusion, and secondary use of personal information [16]. The *perceived benefits* of using TraceTogether were measured with 5 items that assessed the extent to which the participant perceived the health and information benefits they received from using TraceTogether [52]. Finally, *use intention* was measured using three items. Checking TraceTogether was mandatory to enter most public venues. Therefore, the measurement assessed their intention to use TraceTogether voluntarily until the pandemic ended, rather than asking about their intention to use the device within a certain period. All items used a 7-point Likert scale (1=strongly disagree to 7=strongly agree) unless otherwise indicated. Table 2 lists the measurement items and their factor loadings.

**Table 2:** Measurement items and factor loadings.

Variable and items		Values, mean (SD)	Factor loading, β
**Trust in government**		4.80 (1.53)	
	Even if not monitored, I would trust the government to do the right thing.		.91
	I trust the government to protect my personal information.		.96
	I believe that the government is trustworthy.		.95
**Sense of community (sentences complete the phrase “When I use TraceTogether...”**		4.51 (1.62)	
	It makes me feel like I am part of a community.		.89
	I feel a high sense of attachment with other users.		.94
	I feel an emotional connection with other users.		.93
	It reminds me of the people around me.		.90
	It makes me feel a sense of belonging.		.94
**Privacy concerns**		4.71 (1.50)	
	I believe that my location is being monitored in real time when using TraceTogether.		.83
	I am concerned that TraceTogether is collecting too much information about me.		.88
	I am concerned that TraceTogether may monitor my activities.		.91
	I am concerned that my activities are being monitored.		.91
	I feel that as a result of using TraceTogether, others know more about me than I am comfortable with.		.88
	I believe that as a result of using TraceTogether, information about me that I consider private is now more readily available to others than I prefer.		.90
	I feel that as a result of using TraceTogether, information about me is out there that, if used, will invade my privacy.		.90
	I am concerned that TraceTogether may use my personal information for other purposes without notifying me or getting my authorization.		.90
	I am concerned that TraceTogether may use my information for other purposes.		.87
	I am concerned that TraceTogether may share my personal information with other entities without getting my authorization.		.87
**Perceived benefits**		5 (1.39)	
	Using TraceTogether would improve my access to my health information related to COVID-19.		.86
	Using TraceTogether would improve my access to my health information related to COVID-19.		.87
	Using TraceTogether would improve my ability to manage my health.		.93
	Using the COVID app would improve the quality of health care.		.91
	I would manage my health more effectively using TraceTogether.		.90
**TraceTogether use intention**		5.23 (1.40)	
	I will use TraceTogether even if TraceTogether becomes optional when entering public venues.		.86
	I am willing to use TraceTogether until the COVID pandemic ends.		.74
	I plan to use TraceTogether even if I am not monitored.		.86

#### Ethical Considerations

The study procedure and questionnaire were reviewed and approved by Nanyang Technological University Institutional Review Board (IRB-2022-213). A consent form was displayed on the first page of the survey, and the participants were required to provide their consent to complete the study.

#### Results

##### Measurement Model

Confirmatory factor analysis was conducted using Mplus (version 8.8; Muthen & Muthen) statistical software with a maximum likelihood estimation method. The confirmatory factor analysis results indicated a reasonable fit to data: χ^2^_286_=937.9 (*P*<.001), root mean square error of approximation=0.06 (90% CI 0.056-0.065), comparative fit index=0.97, and Tucker-Lewis index=0.96). The indicators reflected their respective latent variables, as evidenced by the high factor loadings. The magnitude of all factor loadings and Cronbach α were equal to or greater than 0.74 and 0.86, respectively. The measurement model showed robust convergent and discriminant validity: composite reliability and average variance extracted (AVE) were greater than 0.70 and 0.50, respectively. The square roots of the AVEs of all observed variables were greater than the intercorrelations between variables. All factors were significantly correlated with each other. See Table 3 for composite reliability, AVE, and Cronbach α, and see Table 4 for the correlation matrix.

**Table 3:** Validity and reliability.

	CR^a^	AVE^b^	Cronbach α
Government trust	0.958	0.885	0.958
Sense of community	0.964	0.844	0.964
Privacy concerns	0.973	0.783	0.974
Perceived benefits	0.952	0.798	0.952
Behavior intention	0.862	0.676	0.859

^a^CR: composite reliability.

^b^AVE: average variance extracted.

**Table 4:** Correlation matrix (Pearson r).

			Government trust		Sense of community		Privacy concerns		Perceived benefits
**Sense of community**									
	*r*	0.63		—^a^		—		—	
	P value	<.001		—		—		—	
**Privacy concerns**									
	*r*	−0.27		−0.17		—		—	
	P value	<.001		<.001		—		—	
**Perceived benefits**									
	*r*	0.48		0.54		−0.17		—	
	P value	<.001		<.001		<.001		—	
**Behavior intention**									
	*r*	0.44		0.47		−0.21		0.63	
	P value	<.001		<.001		<.001		<.001	

^a^Not applicable.

##### Model Testing

The structural equation modeling (SEM) result without the interaction term in the model revealed a good fit: *χ*^2^_562_= 1382.9 (*P*<.001), root mean square error of approximation=0.048 (90% CI 0.045-0.052), comparative fit index=0.957, and Tucker-Lewis index=0.952. As these model fit indices have not been developed for the SEM models assessing latent interaction effects, we used a log-likelihood (−2 LL) ratio test to compare the SEM model where the interaction was not estimated (parsimonious model) with the model where the interaction was estimated (more complex model) [53]. The test was statistically significant, meaning that the model without the interaction term represented a significant loss of fit relative to the more complex model. Thus, we conclude that the model with the interaction term is well-fitted. In addition, the model with the interaction term had lower values of the 2 fit indices, Akaike information criteria and Bayesian information criteria, in comparison to the model without the interaction term.

**Figure 2:**
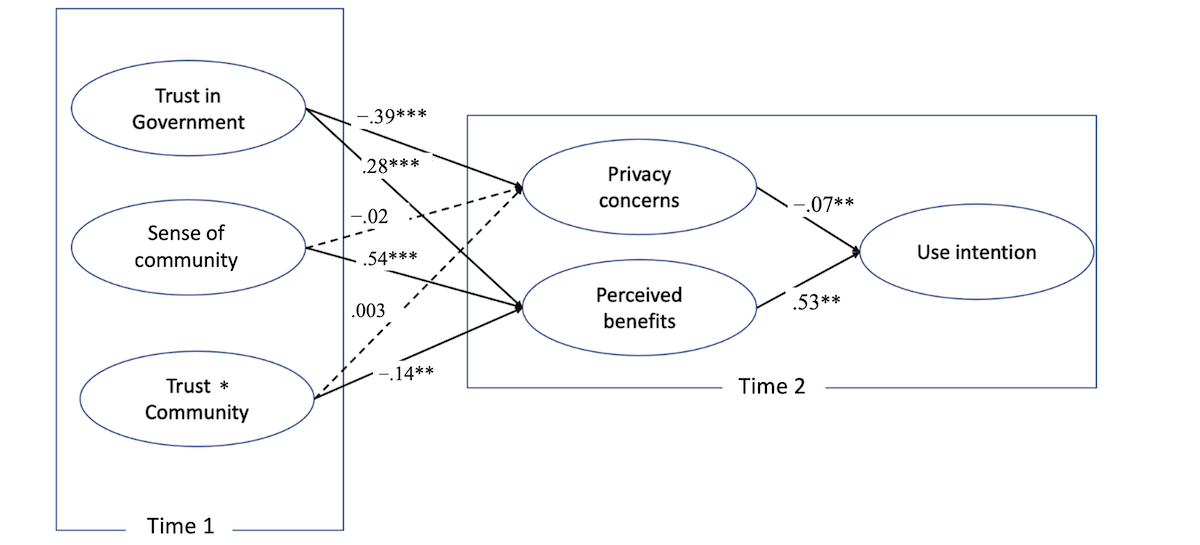
Summary of structural equation model results. *P<.05, **P<.01, ***P<.001. Age, gender, ethnicity, education, income, and use frequency of TraceTogether use measured at time 1 were controlled.

**Figure 3:**
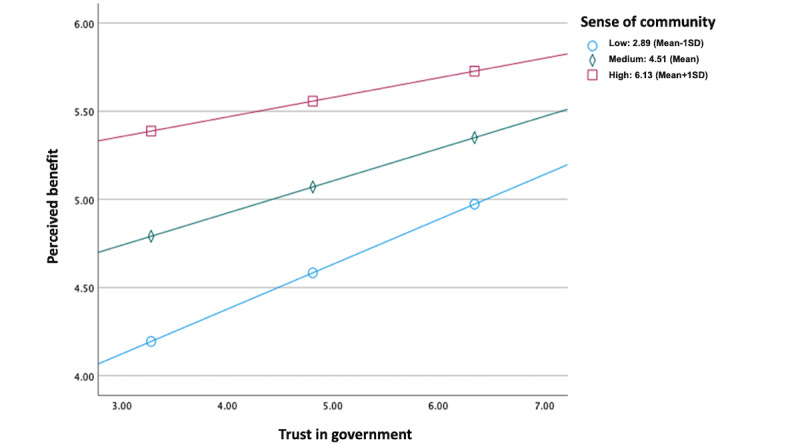
Interaction between the trust in government and sense of community on perceived benefits.

Our analysis revealed that trust in the government significantly reduced privacy concerns of TraceTogether (β=−.39; *P*<.001), whereas sense of community did not have a significant impact on privacy concerns (β=.02; *P*=.86). However, both government trust and sense of community significantly increased the perceived benefits of using TraceTogether (government trust: β=0.28; *P*<.001; sense of community: β=.54; *P*<.001). H1, H2 and H3 were supported, while H 4 was not supported. Regarding RQ1, the results indicated that trust in the government and sense of community interacted only with perceived benefits (β=−.14; *P*=.004). The results are summarized in Figure 2. The interaction pattern (Figure 3) shows that the triggering sense of community has a larger impact on those who display lower trust in the government than on those with high trust. This finding suggests that triggering a sense of community can buffer the negative impact of skepticism in the government on their perceptions of the benefits they can obtain using TraceTogether.

Finally, supporting the privacy calculus hypothesis, privacy concerns decreased TraceTogether use intention (β=−.07; *P*=.04) whereas perceived benefit increased use intention (β=.53; *P*<.001). Hence, H5 and H6 were supported.

### Study 2: After In-Depth Interviews

#### Overview

The survey results illustrated the effects of trust in the government and a sense of community on TraceTogether privacy calculus and, ultimately, TraceTogether users’ intentions. However, with the survey data, we could not identify which aspects of privacy concerns were factored in the privacy calculus, influencing their intention to use TraceTogether. In addition, given the significant roles of trust in government and sense of community in TraceTogether and privacy calculus identified by the survey results, we conducted in-depth interviews to further understand the underlying reasons for these effects. The purpose of this study was two-fold: 1) to understand the reasons underlying the effects of government and sense of community on the privacy calculus of TraceTogether use, and 2) to obtain a deeper understanding of different aspects of privacy concerns among TraceTogether users and the implications for their privacy calculus.

#### Methods

#### Participants and Procedure

To obtain additional insights into the privacy calculus of TraceTogether, we conducted in-depth interviews with 12 Singaporeans between April 13 and May 11, 2022. We recruited Singaporeans (mean age 38, SD 13.28; 7 female participants, 5 male participants; see Table 5) who used TraceTogether during the COVID-19 pandemic. Among them, 7 were Chinese, 3 Malay, and 2 Indian. A trained research assistant conducted the interviews using a web-based conference program (ie, Zoom) in English. Participants were given a link to the consent form before starting the interview, which began once their consent was received. Each interview followed a preconstructed list of questions to understand their perceptions and use of TraceTogether. After completing the 4 interviews, the TraceTogether check-in was no longer used, starting from April 26. Subsequently, for the remaining 8 interviews, the participants were asked to answer questions reflecting their prior experiences with TraceTogether. Interview recruitment was stopped when we reached a state of theoretical saturation [54].

**Table 5:** Demographics of in-depth interview participants.

ID	Gender	Ethnicity	Age (y)	Occupation
1	Male	Indian	24	Student
2	Male	Chinese	23	Student
3	Female	Chinese	23	Student
4	Female	Chinese	49	Manager
5	Male	Chinese	56	Teacher
6	Female	Chinese	49	Teacher
7	Female	Malay	50	Teacher
8	Female	Malay	31	Teacher
9	Male	Malay	31	Clinician
10	Male	Chinese	51	Worker in the Singapore armed forces
11	Female	Chinese	49	Administrator
12	Female	Indian	24	Social media and project manager

#### Data Analysis

We used the 3-step protocol [55], which provides a structured framework for qualitative researchers to systematically analyze and derive meaningful interpretations from their data, involving 3 stages of data analysis: open-coding, axial and hierarchical coding, and theoretical interpretation, outlined as follows: First, 2 research assistants, including the interviewer, reviewed and coded the transcribed interviews independently. They read the transcribed interviews line by line and assigned the initial codes according to the themes of the original conversations (eg, concerns about using TraceTogether and adoption barriers). The second step was the axial and hierarchical coding stage; 2 researchers identified the second-level interpretive themes based on the theoretical framework and hypotheses (eg, surveillance, intrusion, and secondary use of data) by analyzing and grouping the initial coding performed by the 2 research assistants. Prior literature on web-based privacy calculus and privacy issues of digital contact tracing guided this coding stage. Finally, the researchers discussed how to interpret the themes that surfaced in the second stage in order to answer the research questions.

#### Ethical Considerations

Participants were given a link to the consent form before starting the interview, which began once their consent was received. The consent form, interview questions, and protocols were reviewed and approved by the primary investigator’s institute (IRB-2022-213).

#### Results

##### Overview

In a scenario where policies on and implementation of a contact tracing system were rolled out urgently and comprehensively, privacy became a significant barrier to adopting the program. Our study revealed that trust in the government serves as a social lubricant, effectively mitigating privacy concerns arising from the inherent risks associated with the tracing program, thereby facilitating smoother implementation of the program. Our interview results indicated that trust in the government translated into the belief that the government would possess adequate security measures to safeguard citizens’ privacy and prevent the misuse of their information. However, considering other community members while participating in the contact tracing program does not necessarily alleviate privacy concerns. The interviews also highlighted surveillance as the most prevalent aspect of privacy concerns regarding TraceTogether use. In addition, our findings revealed that trust in the government, particularly the perceived transparency of government actions, was most related to concerns regarding the secondary use of data. The detailed results, accompanied by key supporting quotes, are outlined in the following sections.

##### The Role of Trust in Government in TraceTogether Privacy Calculus

The interview responses also suggested a significant influence of trust in the government on both perceived benefits and privacy concerns. Consistent with the findings of our web-based survey, the results indicated that individuals who trust the government were more likely to perceive TraceTogether as beneficial while exhibiting lower privacy concerns. For example, participant 7 (female, age 50 y) did not understand why the app should track user locations; therefore, she felt that she did not want to use it. However, she explained that she downloaded the app because she trusted the government, stating, “I trust that my government knows what they’re doing to the people. I trust that it is for the good of us*.*” Conversely, participant 12 (female, age 24 y), who indicated distrust in the government, did not perceive any additional benefits of using TraceTogether beyond using QR codes for checking-in, stating, “I feel like using the QR code would still work.” Furthermore, she voiced several privacy concerns, remarking, “What if this thing is tracking me 24/7? What if I am at home has my constant location at home, and my address is being transmitted?” She also referenced the public backlash at the start of 2021, when Foreign Minister Vivian Balakrishnan revealed that the police could access TraceTogether data for criminal investigations. This announcement contradicted his statement in June of the previous year that TraceTogether data would only be used for contact tracing [56]. She mentioned that she felt betrayed by government. Regarding the same incident, however, participant 5 (male, age 56 y) felt indifferent, stating: “There’s nothing to hide, so I am not concerned. Those who are criminals, they should be afraid. Good for them. They are better afraid*.*” His indifference was his strong trust in the government. He added, “I think the government is smart enough not to use it against themselves, because if it blows out of proportion, I tell you, the government will pay a high price. I don’t think they try to do that*.*”

##### The Role of Sense of Community in TraceTogether Privacy Calculus

Our survey data revealed that a sense of community when using TraceTogether significantly increased the perceived benefit but did not influence their privacy concerns. The interview respondents also understood that collective efforts were required to make the digital contact tracing system successful. For example, participant 1 (male, age 24 y) mentioned, “If enough people download it and contact tracing becomes easier, then we can, in a sense, fight this disease and end it earlier instead of letting it prolong.” The responses suggest that protecting the community is a strong motivation for adopting a digital contact tracing system. However, consistent with the survey results, the interview responses indicated that a sense of community did not necessarily alleviate concerns about privacy invasion through the contact tracing system. Participant 5 (male, age 56 y) stated, “It’s for the greater good. If it’s going to protect the country from this virus, it would be good. It won’t last forever. These two years, you have had your privacy invaded, but subsequently, you are fine.”

##### Dissecting the Multiple Aspects of Privacy Concerns in TraceTogether

As discussed earlier, privacy concerns are not a single-faceted concept, but encompass multiple aspects, including perceived surveillance, perceived intrusion, and secondary use of data [16]. Through the postinterviews, we attempted to understand the different aspects of privacy concerns among TraceTogether users.

Surveillance stood out as the most prominent privacy concern, as mentioned by 7 of 12 respondents. Respondents explained that lack of control contributes to their fear and anxiety about being watched, even if they feel that they have nothing to hide. Some respondents preferred using a TraceTogether token to the mobile app to reclaim a sense of control over where and when their locations were traced. For example, participant 7 (female, age 50 y) mentioned, “I am not afraid because I don’t have anything to hide and that I am doing anything wrong or I am a criminal... It’s just that... I don’t like to know that my personal particulars are being shared.” Participant 4 (female, age 49 y) also shared, “I can put it [token] at home...They cannot even trace me to home. They can trace the data on my phone.” These deliberate choices can be understood as small acts of resistance to mandated surveillance.

The respondents also shared that the digital contact tracing system, in fact, collected more information beyond what was necessary, such as how participant 4 (female, age 49 y) expressed her concerns. “Government is going to trace me where I go, what I do, who I meet. I feel very invaded by my privacy.” From the responses, we discovered that perceived intrusion becomes prominent because they do not know what information is to be tracked and used and the purpose of tracking clearly. Participant 2 (male, age 23 y) said, “I never know what they use the information for... They want to check whether we stay at home. It’s still not right because it’s an invasion of privacy.” Participant 4 (female, age 49 y) also raised concerns that TraceTogether would gather information beyond what has been told: “[if] I go to the shopping mall, and then they start to want to know how much you spend and say how much you spend in that shopping mall or what exact activities are being conducted.”

In addition, several respondents cited distrust in the government as a factor that increased privacy concerns, specifically with regard to the secondary use of data. Regarding the government’s announcement that the police could use TraceTogether data for criminal investigations, participant 3 (female, age 23 y) expressed her concerns, saying, “I thought it was a bit annoying because there was a lack of transparency about whatever they were going to use the app. If they wanted to do it for this stuff, they should have said it earlier and not let it be like, ‘Oops, I said it as an accident.’” The government’s transparency was revealed as an important factor in determining users’ privacy concerns about the secondary use of data. Transparency on how contact tracing apps work can not only affect user confidence, but also determine ethical considerations. Participant 2 (male, age 23 y) shared his point, stating, “If they ask for your consent and you decide to give it (tracked information), then sure. If they do it on their own, then that’s not okay.” Participant 1 (male, age 24 y) shared a similar point, explaining: “It’s just that I’m perfectly fine with them in using this app for in terms of national security or intelligence or what, but what I want them to do is be upfront and open.”

## Discussion

### Theoretical Implications

Using a mixed method approach involving a 2-wave survey and in-depth interview data, we expanded our understanding of privacy decisions and calculus in the context of digital contact tracing. Our study has several notable findings. First, we found that privacy concerns had a marginal negative association with use intention, whereas perceived benefits had a strong positive association, consistent with previous research [57,58]. The opposite influences of privacy concerns and perceived benefit on use intention suggest that the privacy calculus in TraceTogether might be viewed as a rational process of weighing privacy risks and use benefits to make an uptake decision. Second, although our findings on the relationships between trust in government and sense of community and privacy calculus are largely consistent with existing research [59], our study demonstrated that their existing perceptions toward the provider and the government in the contact tracing context, as well as the perception of the community triggered by TraceTogether use, may bias user appraisals of privacy risks and benefits of contact tracing. Finally, our research extends the existing knowledge of privacy calculus by delineating how the sense of community moderated the relationship between perceived benefit and trust in government, such that individuals’ sense of community elevated perceived benefit, even for those with low trust in government. The study results provides several important theoretical implications: First, the study illuminated how people’s trust in the system provider plays a role in privacy calculus in digital contact tracing systems. The digital contact tracing system, including TraceTogether, is a special form of a web-based system built and managed by government organizations during an unprecedented pandemic. Digital contact tracing entails delegating the right to track and process location, contact, and health-related information to the government’s discretion. Therefore, we posit that trust in government is a powerful factor in determining social exchange decisions.

As expected, trust in the government significantly influenced privacy calculus at the later stage when the use of TraceTogether became mandatory for entering most venues and workplaces. The results support prior research, which suggests the importance of trust in the government for the successful implementation of the contact tracing program for infectious disease prevention (eg, [32-36]). Trust in the government becomes pivotal in government-citizen relationships, especially during the implementation of new policies. This is largely because trust can reduce the transaction costs in relationships [60]. Our interview responses offer deeper insights into how trust in the government lowers transaction costs, specifically addressing privacy risks in the context of participating in the contact tracing program. We found that one’s privacy calculus can be biased according to the level of trust displayed by individuals, and an interviewee showing distrust in the government tends to exaggerate the privacy risk of TraceTogether while doubting its effectiveness without specific evidence. Conversely, another interviewee, who started to use TraceTogether without knowing its purpose of tracking one’s location, shared that she downloaded the app simply because she trusted the government, and thus trusted that the app was good for them.

Our results also revealed that the sense of community elevated by TraceTogether use positively influenced users’ benefit appraisal, even though it did not reduce privacy concerns. This finding is in line with research [61], which found that in largely collectivistic societies (eg, China), there is a greater acceptance of contact tracing apps compared with more individualistic societies (eg, the United States, Germany), and people are generally willing to accept these technologies, especially if they are considered effective. In our study, the results might be because Singaporeans accept the spirit of the “greater good” underlying the contact tracing measure in the pandemic situation in the community, supporting the extant literature. A prior study compared Singapore and Switzerland and attributed the former’s higher rates of acceptance and adoption to its norms that prioritize the “interests of the community” [46]. Interestingly, privacy concerns regarding the use of TraceTogether persisted even when a sense of community was fostered. The follow-up interviews revealed these findings. For instance, our interviews indicated that considering fellow community members while participating in the contact tracing program motivated individuals to endure the discomfort of sacrificing privacy for the greater good rather than directly alleviating the concerns themselves. This aligns with prior studies on the role of sense of community in coping behaviors; they showed that a stronger sense of community motivates individuals to focus on problem-solving coping behaviors [62,63]. By extending these findings to our study, it can be inferred that strengthening the sense of community compels individuals to concentrate on addressing the larger community issue while accepting potential risks associated with participating in the program to tackle this issue.

Our postinterview responses provided an in-depth understanding of privacy calculus in TraceTogether use by dissecting different aspects of privacy concerns [16] that TraceTogether users exhibited. The interviewees responded that they felt they were being surveyed through TraceTogether, which often required excessive personal information. However, interviewees were generally willing to bear surveillance and intrusion for the greater good of the community and their own safety. However, the results indicated that having a sense of control over information is an important factor in the privacy calculus in TraceTogether. Most interviewees also called for greater transparency in data collection and secondary use of data. They actively sought strategies to better control their personal information. For example, some participants indicated that they prefer to use the token rather than the app so that they can take the token only when TraceTogether checking-in is required. Hence, our study results support the notion that privacy management is a dynamic process involving “*selective control* of access to the self or to one’s group” [64].

Another notable finding of our study was how the sense of community could elevate the benefits of contact tracing despite low trust in the government. This is important considering that norms-based approaches [65] are effective in modifying health behaviors in diverse contexts. Existing research has identified several moderating factors that influence users’ intention to use contact tracing apps. For instance, demographic factors, such as gender, racial and ethnic identity, and education level, are significant moderators of contact tracing app design on people’s intention to install apps [66]. Separately, another study found that self-efficacy was a significant moderator of trust and intention to use contact tracing apps [67]. Although these findings are important for technology developers, they view technology use and acceptance through a largely technocentric and individualistic approach, even though public health scholars [68] have argued for the need to consider societal factors as driving factors of adoption, as evidenced by research [69] on how norms could drive app use, even among marginalized communities.

### Practical Implications

Our study offers valuable insights into the policy and design considerations concerning digital contact tracing programs. The interview results revealed that, although Singaporeans are willing to accept a certain level of surveillance for preventive measures via contact tracing, issues arise when data use lacks transparency, potentially causing individuals to reconsider their positive intentions to participate in the greater good. Moreover, because digital contact tracing was made mandatory, they also felt that they had no control over their information, which may overturn their privacy calculus, and thus opted out from participating in the program if it became optional. The results suggest that for the successful implementation of the contact tracing program, it is crucial to implement policy measures to ensure clear and transparent communication regarding the use of data collected from contact tracing practices. In addition, providing as many control options as possible would encourage users to maintain active participation in the contact tracing program.

The moderating effect of a sense of community prompted by contact tracing has valuable practical implications. The interaction result indicated that the negative impact of distrust in government on contact tracing program participation can be overcome by promoting the “social good” nature of the contact tracing program via campaign or application design. According to our study results, the campaign for TraceTogether that framed contact tracing as a community effort and responsibility, with the campaign slogan being “Protect your community,” might have enhanced community participation, especially among those with lower trust in the government. If, unfortunately, another pandemic emerges in the future, and a similar program is needed, the contact tracing program can actively incorporate social- and community-related features. For example, in the early stage of implementation, the application can be promoted through social networks, allowing users to invite and encourage their family and friends to opt for the program.

Additionally, the contact tracing app interface can be designed to cue a sense of community when using it. Illustrations of the family and community for the application interface design, as TraceTogether has done, would be an effective strategy to enhance participation in the program. TraceTogether also displays the number of Bluetooth signals shared by others. However, such information can be framed as emphasizing collective efforts for the greater good of the community. For example, information can be shown through a visual illustration of people forming networks.

### Limitation and Future Research Direction

This study had some limitations. Although the longitudinal survey design allowed us to establish the temporal sequencing of the variables of interest, the dependent variable (ie, behavioral intention) was assessed in the follow-up survey without obtaining the baseline measurement. Hence, we cannot fully leverage the longitudinal nature of the data to establish the direction of the association or causal relationships between the variables. Future studies may use longitudinal data with repeated measures of the variables (two or more time points) to address them. Additionally, the data provide limited potential for generalizability. Survey participants with a higher socioeconomic status (eg, education) were overrepresented. In addition, the use of self-reported data in surveys may pose the risk of social desirability and recall biases. Finally, we acknowledge that the mandatory use of TraceTogether between waves 1 and 2 of our survey might have an impact on the use intention of participants, as existing research [70] on a Singapore sample showed high adoption due to the mandatory use of the app for entering public venues. To circumvent this, we modified our items such that they captured participants’ volitional use in contexts when TraceTogether became voluntary, rather than capturing actual use behavior as the dependent variable due to social desirability bias. Despite the above limitations, we are confident that this study has presented valid evidence of the relationships among trust, privacy concerns, perceived benefits, and TraceTogether use intentions.

## Abbreviations

AVE: average variance extracted
SEM: structural equation modeling
